# Impact of Training Discipline and Experience on Inhibitory Control and Cognitive Performance in Pet Dogs

**DOI:** 10.3390/ani14030428

**Published:** 2024-01-29

**Authors:** Nerys Mellor, Sebastian McBride, Emma Stoker, Sarah Dalesman

**Affiliations:** 1 Department of Life Sciences, Aberystwyth University, Aberystwyth SY23 3DA, UK; nerysmellor05@gmail.com (N.M.); sdm@aber.ac.uk (S.M.); 2 Puppy Plus Training and Behaviour Services, Newtown SY16 3HQ, UK; puppy.plus@yahoo.co.uk

**Keywords:** A-not-B task, canine, cognition, detour task, domestic dogs, impulsivity, inhibitory control, persistence

## Abstract

**Simple Summary:**

Training experience has been shown to have positive impacts on the behaviour of dogs when solving cognitive tasks, but previous work has focused on comparing highly trained working and sporting dogs with untrained pet dogs. Here, we assessed whether training a pet dog in scent work, agility, and obedience can also improve the dog’s ability in two tasks: (1) the A-not-B task, in which a dog first learns to go to a pot on one side of a three-pot lineup to obtain a reward and then must change this behaviour to go to the pot on the opposite side; and (2) the detour task, in which a dog must navigate around a transparent barrier to obtain a visible reward. Dogs that performed better on the A-not-B task were also more likely to be successful in the detour task, showing that there is a relationship between the skills required to perform well in both tasks. Training did not affect overall performance in either task, but dogs trained in scent work showed better inhibitory control across the two tasks. This indicates that scent training for pet dogs may be beneficial in improving behaviours that require inhibition.

**Abstract:**

Training experience has been shown to enhance a dog’s cognitive performance when comparing highly trained working or sporting dogs with untrained dogs. However, whether the type or level of training a pet dog receives can alter their performance in cognitive tasks requiring inhibitory control has not been assessed. Here, we tested whether pet dogs trained in scent work, agility, and obedience differ in cognitive performance. The impact of primary training discipline and combined training experience was assessed using two well-defined tasks that require inhibitory control: (1) the A-not-B task, in which dogs must inhibit a previously learned response in favour of an alternative response; and (2) the detour task, in which dogs must inhibit a direct approach to food to gain a reward. Dogs trained in scent work demonstrated higher levels of inhibitory control and persistence across the two tasks, but this did not affect individual task performance. Increased combined training experience improved learning in the A-not-B task training phase, but did not alter performance during the test phase, whereas it had no effect on success in the detour task. Overall, dogs that performed better in the A-not-B task were also more likely to succeed in the detour task, showing a relationship in the cognitive ability underpinning performance in the two tasks. The effect of the primary discipline on the behavioural phenotype shows that this should be accounted for in future studies, rather than applying the practice of partitioning dogs into highly trained vs. untrained groups.

## 1. Introduction

In modern society dogs (*Canis lupus familiaris*) have to perform a wide range of roles including assistance [1], detection [2], herding and guarding livestock [3] and competitive sports [4], as well as companionship as pets [5]. The trainability and cognitive performance of these dogs in each of their different roles is highly dependent on a number of factors that include genetics (breed), level of training, motivation, arousal, inhibitory control, persistence, human dependence, and independence in problem solving, and these factors may also interact with one another in different ways [6,7,8,9]. Breed can influence how dogs interact with humans; for example, pastoral breeds show a stronger preference for looking to their owner for help than do gundog breeds [10,11], and working dog breeds are better at understanding human gestures than non-working breeds [12], but experience can overcome breed differences [13]. This indicates that training and experience may be just as important as genetics in determining the cognitive performance of dogs [14].

Studies on the impact of training background (discipline and training level) on problem solving ability have shown differences between dogs trained to a high level in working or sporting roles and pet dogs that have received little or no training. For example, dogs that are highly trained for work or sporting activities outperformed dogs with only basic obedience training in a box opening task [15], as well as in both a detour and a puzzle box task [16]. Search and rescue dogs outperformed pet dogs with basic obedience training in an A-not-B task, showing higher levels of inhibitory control [6], and improved performance in a puzzle box task [17]. Trained assistance dogs outperform pet dogs in a detour task [7] and outperform pet dogs with recreational sport training (primarily obedience and agility) or no training on a puzzle task [18], although in the latter study, dogs trained in a recreational sport also outperform those with no training.

Inhibitory control has been shown to have a significant effect on canine problem solving ability [19]. This describes the ability to inhibit an impulsive action, immediately favourable but undesired or inappropriate, in favour of an alternative action, typically more desired and appropriate for the situation [6,20]. Highly trained dogs demonstrate sustained attention to stimuli [21] and enhanced levels of inhibitory control compared to untrained dogs [6,7,16]. Inhibitory control involves complex cognitive processes and may be influenced by motivation and arousal, and good inhibitory control is beneficial in tasks requiring dogs to ignore stimuli in their environment or work in high arousal environments. 

Highly trained dogs exhibit more motivation and persistence in problem-solving tasks than do untrained dogs [16,17]. Training can also alter the level of dependence on cues from the handler, as working dogs appear to be less socially dependent on their owners [18,22]. These studies primarily focus on training level, rather than on the impact of specific discipline; however, training disciplines that require different cognitive skills are predicted to impact on the development of executive function [18,23], which may in turn affect problem solving ability. 

Independence is also a critical factor in determining trainability and cognitive performance. Scent detection dogs, for example, are required to act independently when performing scent detection work [24] and also have to exhibit good task focus, requiring some level of inhibitory control to avoid distraction from a search [25,26]. Agility dogs are required to follow their handler’s cues and signals throughout agility training and performance and demonstrate a higher level of handler gaze during problem solving compared to untrained pet dogs [27,28]. Obedience dogs show even less independent thinking while performing their specific discipline, as they are required to closely follow each command given by their handler, although these dogs must also display a good level of inhibitory control to maintain focus on their handler [14,29]. 

Additional factors that also require consideration in determining the trainability and cognitive ability of dogs include sex, age, temperament, and arousal. In a study involving 483 males and 549 females, female dogs achieved a higher success rate in the cylinder task than male dogs, indicating that females have greater inhibitory control [30]. Age has been found to influence inhibitory control, which decreases in older dogs, consequently exerting negative effects on cognitive ability [20] and cognitive flexibility [31]. Temperament can also affect the reliance of dogs on the owner vs. an unfamiliar demonstrator [9]; dogs with ‘irritable’ behaviours, as reported by owners, gazed less towards their owner during a detour task, but performed better when the task was first demonstrated by an unfamiliar person. Increased arousal can enhance performance, but if an animal is already in a higher arousal state, further arousal is likely to result in poorer cognitive performance [32]. In pet dogs, higher levels of arousal typically have negative impacts on performance when compared with the performance of highly trained working dogs [7]. 

Considering these primary factors affecting trainability and cognitive performance, the aim of this study was to assess the effect of training discipline (obedience, scent work, and agility) and training level on problem solving in pet dogs, using two specific cognitive tasks (the A-not-B task and the detour task) that are heavily reliant on inhibitory control for their successful completion [33,34]. The A-not-B task determines whether the dog is able to inhibit search behaviour towards a previously rewarded location after watching the experimenter move the reward to a new location [20,35,36]. In the detour task, the dog must move around a transparent barrier to access a visible reward, requiring the dog to inhibit its direct approach response to the reward [7,34]. We therefore predicted *a priori* that scent work dogs, who are required to work independently and who require inhibitory control to remain task focused during searches, would outperform dogs primarily trained in the other disciplines, and that they would demonstrate enhanced performance in both the A-not-B and detour tasks. In addition, we also assessed dogs for stress indicators and eye contact with their handler as measures of anxiety, arousal level, human dependence, and attachment [7,37,38]. We predicted that dogs showing a high number of stress indicators and increased eye contact with their handler would demonstrate poorer cognitive performance due to high arousal and lack of handler independence.

## 2. Materials and Methods

### 2.1. Recruitment and Dog Background Information

Study recruitment was carried out via an online questionnaire posted in social media groups run by local trainers and dog communities. This survey included consent for the researchers to retain the owners’ contact information for the duration of the experimental period and consent of the owners who completed the survey to be contacted to participate in the trials. Owners who had agreed to be contacted received an information sheet outlining what to expect, without detailing the protocol, and a participant consent form which they had to sign prior to taking part. The survey, owner information sheet, consent form, and data storage protocol were reviewed and approved through the Aberystwyth University Human Ethics Approval procedure. Data were stored on a secure drive, and all personally identifying information was permanently deleted at the end of data collection.

The questionnaire asked owners to indicate the level of training that their dog participated in across three disciplines: obedience, scent work (competition and man trailing), and agility (see Supplementary Material: Abbreviated Survey). These were chosen as the three most common training activities in which dogs owners participate, based on a preliminary survey of local training clubs. The levels for each training activity are outlined in Table 1 and were based on competition levels and relative difficulty, as judged by experienced local trainers and competitors in each activity. The survey included descriptions of each level so that participants who were unfamiliar with the guidelines were able to judge the level to which they should assign their dogs. For the purposes of analysis, scent work was grouped as a single discipline, with the highest score across competition scent work and man trailing allocated as the participants’ score in the scent work category. The primary training discipline was identified by determining the training discipline in which an individual had achieved the highest training level. The combined training level was calculated by adding together the training level score from an individual’s primary discipline with the training level score obtained in other disciplines if the individual engaged in multiple disciplines. Background information was also gathered, including the sex, neuter status, breed, and age category of the dogs (<12 months, 1–2 years, 3–4 years, 5–6 years, 6–8 years, and >8 years). Breed group was based on the owner’s description of their dog in the survey and verified visually by the experimenter. Breed was classified into three groups, based on British Kennel Club classification: gundogs and their crosses, pastoral breeds and their crosses, and ‘other’, which included a mix of breeds from other groups (See Supplementary Table S1 for breed descriptions).

### 2.2. Dog Participants

A total of 40 dogs were invited to participate in the trials (Table 2). The training level across three disciplines was used in the assessment of the effect of training on performance, obedience, scent work, and agility. A total of 4 dogs did not attend training classes, 10 dogs only trained in a single discipline, 19 trained in two disciplines, and 7 dogs trained in all three disciplines. The primary training discipline was based on the relative skill level across the three disciplines, with the discipline demonstrating the highest skill level considered the primary discipline. Of the dogs that had attended training classes, 17 were primarily trained in obedience, 11 in scent work, and 8 in agility (Table 2).

### 2.3. Ethical Statement

The experimental protocol did not fall under ‘Scientific Procedures’, as determined by the Home Office, U.K., so no licence was required to carry out the work. The experimental protocol was reviewed and approved by the Aberystwyth University Animal Welfare and Ethical Review Body (AWERB, protocol ID code 118, date of approval 17 October 2022). Potential participants were screened to remove any dogs with a history of aggression towards humans. Participants were also asked to declare whether they or their dogs had any known allergies, and if the dog had any history of dog reactivity. The latter possibility was accounted for when assigning dogs to time slots to ensure no overlap with other dogs. The experimenter was trained in the recognition of dog stress signals and stopped the experiments if dogs demonstrated anything considered above mild uneasiness with the testing environment. Owners were also instructed that they could end their dog’s participation at any point during the trials if they felt their dog was not comfortable with continuing. Dogs were given short breaks (10 min) between each element of the experiment, with free access to toileting and water. 

### 2.4. Experimental Protocol

The owners stayed with their dogs during all research trials and were provided with clear instructions on how to behave and interact with their dog during each task. All dogs were video recorded on a Sony Handycam HDR-CX405 during the behaviour assessment and cognitive tasks; behavioral measures used in analyses were recorded from the video.

#### 2.4.1. Eye Contact and Stress Indicators

On first entering the task area, owners were required to stand in place with their dog on a loose lead, ignoring their dogs (no verbal commands, no dog-directed gazing) for 60 s during an initial observation period. Videos were analysed for behavioural measures. These measures consisted of recording the total amount of time, in seconds, that a subject gazed directly at their human handler as a measure of owner attachment [38], as well as the frequency of stress indicators performed, which included lip licking, yawning, and vocalisations such as barking or whining [39,40]. Sniffing behaviour can also be a displacement activity and an indicator of stress [41,42]; however, as we would also expect dogs to explore a new environment using olfaction, we did not include this as a stress indicator. 

#### 2.4.2. A-Not-B Task

**Figure 1:**
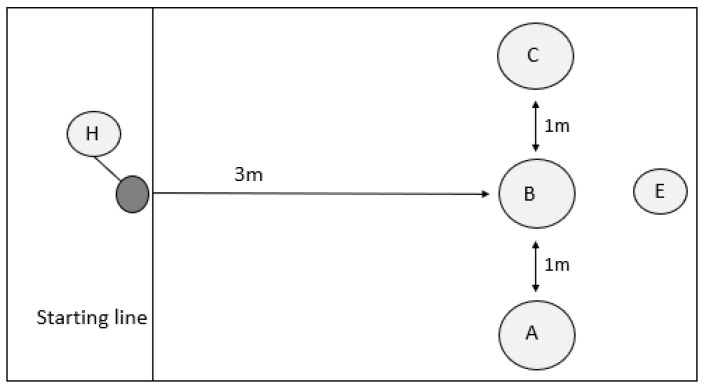
A-not-B task experimental design. The experimenter (E) baits pots in position A, B, or C in familiarisation trials; pot A or C in the training trials; and either A or C (opposite pot) in the test trials. The experimenter moves to position E and turns their back on the handler and dog, indicating ready. The handler (H) releases the dog to enable it to identify the food location and obtain the food reward.

White indoor plant pot covers, measuring 21cm height x 18 cm diameter (Whitefurze, Coventry, UK), were used in the A-not-B task. JR pet pate (JR Pet Products, Brecon, UK) was used as the food reward. All pots used in the trials were scented with pate by rubbing a small portion vigorously around the inside rim of the pots to prevent dogs from being able to discriminate the rewarded pot using odour. At the start of each trial, the owners were asked to loosely restrain their dog behind the start line (Figure 1, position H). The experimenter would then state ‘look’ in a neutral tone to gain the dog’s attention and place the food under the designated pot (Figure 1: positions A–C), move to position (Figure 1; position E), then turn their back on the handler and dog [35], giving the handler the indicator ‘ready’ to release their dog. The handler would then let go of the dog and use their release word if needed to let the dog know they could move. Handlers were instructed not to use any commands that prompted the dog to search. If the dog failed to perform a motor response within 20 s of being released, or after failure to interact with correct pot, the dog was shown, but did not obtain, the food reward, and the trial was repeated.

**Familiarisation phase:** Each subject was required to successfully complete five consecutive familiarisation trials before proceeding onto the training trials, with a maximum of 20 possible trials. In familiarisation trials, only a single pot was used, but the location of the pot was moved between each trial in a pseudorandom order to ensure that the pot was in each position at least once during this phase (Figure 1: A–C). The handler restrained the dog behind the starting line while the experimenter stated ‘look’ in a neutral tone before placing a baited pot in one of three available locations (A, B, or C) in view of the dog. The experimenter then stood in the middle (Figure 1: position E) location with their back turned towards the dog and indicated that the dog could be released. The handler then released the dog, which was required to interact with the pot to obtain a food reward. Only dogs that succeeded in the familiarisation phase progressed to training.

**Training phase:** Prior to experimentation, dogs were randomly allocated to the ‘A’ baited pot being in either position A or C (Figure 1); the baited pot then remained in the same location throughout. The training trials follow the same procedure as those for the familiarisation trials in that the handler restrains the dog behind the starting line while the experimenter states ‘look’ and then baits a single pot, in location A or C. During this phase, un-baited but scented pots were also available in locations B, and A or C (Figure 1). Again, the experimenter turned their back to the subject before the dog was released by the handler. The dog only obtained a food reward if the first pot they interacted with was the baited pot. If the dog approached the wrong pot first, then the dog was shown, but did not receive, the food reward before being recalled by the handler to repeat the failed trial. Each subject was required to successfully complete five consecutive training trials before proceeding onto the test trials, with a maximum of 20 possible trials. Previous studies have used either three consecutive trials [20] or five correct trials, without requiring them to be consecutive [43]; however, we wanted our test to be more demanding in determining behavioural flexibility.

**Test phase:** During the test phase, the experimenter sham baited the previously rewarded location in the training phase before moving the reward, in full view of the dog, to the opposite location, e.g., if pot A was baited during the training phase, pot A was sham baited, and then the food reward was moved to pot C. The experimenter stated ‘look’ prior to sham baiting the first pot, but did not attempt to gain the dog’s attention again while the reward was being moved. As during familiarisation and training trials, the experimenter turned their back before indicating to the handler to release the dog. To meet the test criteria, subjects needed to select the correct baited pot in at least 8/10 trials to be considered successful, based on an average 80% success rate found in a similar study [20]. Perseverative search errors, in which the subject fails to adjust its behaviour when the rules change (e.g., subject searches the previously baited location ‘A’ or location ‘B’, as opposed to the correct location ‘C’) and choice latency (time in seconds between the dog crossing the start line and the dog choosing a pot) were recorded from the video footage for analysis.

#### 2.4.3. Detour Task

**Figure 2:**
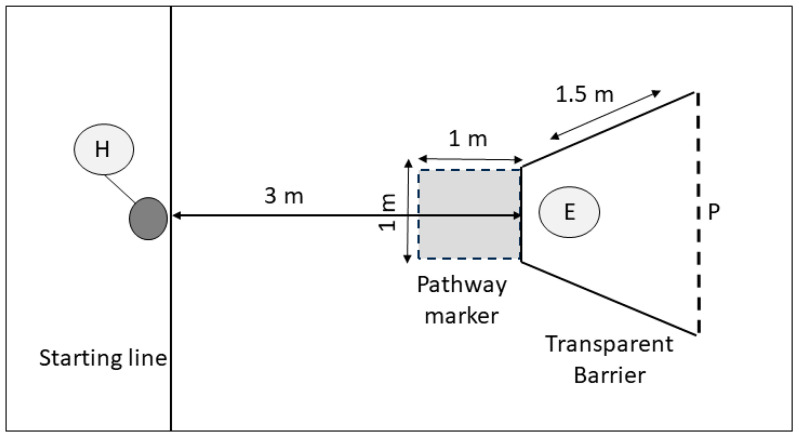
Detour task experimental design. The experimenter (E) holds the bowl containing food reward behind barrier. The handler (H) releases the dog to detour around the barrier and obtain the food reward from experimenter (E). The shaded area indicates the indirect pathway area (path error). P = the line the dog must cross in order to receive the reward.

A transparent barrier was constructed for the detour task using clear heavy-duty tarpaulin (FoundGo, Shanghai, China) on a white UPVC frame using 22 mm FloPlast piping (FloPlast, Sittingbourne, UK). The front of the barrier measured 1 m across, and sides measured 1.5 m in length and 1 m in height (Figure 2). The front of the barrier was placed 3 m away from the start line, and a ‘path error’ zone, 1 m by 1 m, was marked on the floor in tape, directly in front of the barrier (see Figure 2: pathway marker). The path error zone marked the region that the dogs would cross if they approached the barrier directly towards the reward, rather than by taking the shortest route to navigate around the barrier. When in place, the experimenter positioned themself within the barrier, 0.5 m behind the front partition, holding the baited food bowl in front of them (Figure 2: position E).

**Familiarisation phase:** To ensure that the subjects were motivated to approach the experimenter to obtain food from the bowl, a familiarisation phase was carried out without the detour barrier in place. The handler restrained the subject at the starting line while the experimenter kneeled in place at the location they would be when the detour barrier was in place (distance of 3.5 m away). The experimenter placed a food reward in a plastic dog bowl in view of the dog and then called it once, using its name in a neutral, monotone voice. The handler then waited 3 s and released the dog using a release verbal cue, if needed, at which point the dog was required to approach the experimenter and bowl to obtain the food reward. The dogs were required to pass this stage, approaching the experimenter to gain the food reward five times in a row before they were able to progress to the next stage. If the dog failed to approach the experimenter five times in a row over a maximum of ten trials, it was excluded from the task.

The handlers were then asked to step outside the room with their subjects while the detour barrier apparatus was moved into position. The subjects then returned and performed one barrier familiarisation trial during which the handler walked their dog around the detour barrier apparatus on leash, without the experimenter in place, ensuring that the subject could perform the required motor response for the task.

**Test phase:** Test trials required the handler to restrain the subject behind the starting line (Figure 2: position H) while the experimenter knelt inside the detour frame apparatus 0.5 m away from the barrier, facing the dog and handler (Figure 2, position E). The experimenter then visibly placed a food reward into the plastic bowl used during the food familiarisation trials before calling the subject by name once in neutral, monotone voice. The handler was then required to wait 3 s before releasing their dog, using a verbal release cue, if needed. The experimenter remained facing forwards towards the front of the barrier until the dog had successfully navigated the barrier. Failure to perform a motor response within 20 s of release, or an inability to detour the apparatus within a maximum trial length of one minute, resulted in a failed trial. Trials were also labelled as a ‘fail’ if the dog entered the path error zone (Figure 2: pathway marker) immediately in front of the barrier (path error), or made contact with the barrier (contact error) at any point [7]. Once the dogs had navigated past the rear edge of the barrier to the open side and entered the experimenter area (Figure 2: nose crossed line P), the experimenter turned to offer the food reward to the dog. 

Data were recorded from videos of the trials. If the dog navigated around the barrier without making any path or contact errors in 8/10 trials, i.e., taking a direct route, it was considered to have succeeded, providing a measure of success similar to that in the A-not-B task. Path errors (the number of times the dog entered the path error zone) and contact errors (the number of times the dog contacted the barrier) were recorded for each trial. The time taken to navigate the barrier, i.e., the time, in seconds, between crossing the start line and crossing line P, was also recorded for analysis. 

### 2.5. Data Analysis

A total of 40 dogs were invited to participate in the trials (Table 2). Two of the dogs were over-stimulated by the environment and did not engage during the familiarisation periods for either task, so were excluded from all analyses (Table 2: dog ID #15 and #16). Of the remaining 38 dogs, equipment failure resulted in a lack of eye contact and stress indicator data for three of the participants (Table 2: dog ID #1–3), so only 35 dogs are included in the assessment of these behaviours. These three dogs were included in other analyses. One dog was excluded from the A-not-B data analysis, as it failed to engage with the one-pot familiarisation step (Table 2: dog ID #10). Therefore, a total of N = 37 were included for the A-not-B task and N = 38 were included for detour task analyses, except where eye contact/stress indicators were analysed, N = 34 for the A-not-B and N = 35 for the detour task analyses. Dogs were withdrawn if they indicated frustration or stress during either of the test phases. Six dogs that completed training did not complete all 10 test trials in the A-not-B task, and seven dogs did not complete all 10 trials in the detour task. These dogs were included in the analyses of training background, behaviour, and background information as ‘failed to reach criteria’ for each test, but were excluded when assessing performance time or error rate across the trials (see below). 

#### 2.5.1. Analysis of Success Rates

To determine whether the success rate in one task was related to the success rate in the alternative task, generalized linear models were used to analyse both binary response data (pass/fail criteria) and success rate (number of successful trials) in the A-not-B and detour tasks to determine the relationship between A-not-B performance and detour performance and to analyse the effects of age group, sex, neuter status, and primary training discipline. The age group was included in the models as a covariate, and other factors were included as fixed factors.

The relationships between pass/fail criteria and eye contact with the owner, stress indicators, number of training disciplines, and combined training level were analysed using success or failure (and failure during training for A-not-B) as the predictor. Levene’s test for homogeneity of variance was carried out prior to the analyses; if data demonstrated homogeneity of variance, ANOVA was used, with the Welch test replacing this, where required. When homogeneity of variance was confirmed, Sidak post hoc corrections were used; if not, Games–Howell post hoc corrections were used. T-tests were used when comparing two groups based on success/failure for error rates. 

#### 2.5.2. Analysis of Behaviour, Training Level, and Error Rate

Correlations were used to determine whether the number of stress indicators, the eye contact with the owner, the number of disciplines, and the combined training experience were related to performance indicators during the A-not-B and detour tasks. In the detour task, we assessed the relationship between these measures and successful trials, total contact errors, total path errors, and total number of errors across 10 trials. In the A-not-B task, we assessed the relationship between these measures and successful trails and the number of perseverative errors made across 10 trials. Correlations were also used to determine whether the error rate in the A-not-B task related to the error rates in the detour task, and whether different types of error rate related to the success rate in the detour task. 

#### 2.5.3. Analysis of Performance Time

Repeated measures ANOVA was used to determine if the primary training discipline or success in the task affected the speed of performance across trials 1 to 10 for both the A-not-B task (time to first choice) and detour task (time to successfully detour the barrier). The Greenhouse–Geisser correction was used if sphericity could not be assumed. Correlations were also used to determine whether the average speed of performance was related to the error rate in the two tasks.

#### 2.5.4. PCA Analysis

We conducted a PCA analysis on the eye contact time, stress indicators, and the response factors derived from both A-not-B and the detour tasks to determine whether there were underlying differences in behaviours among the participants. The analysis included the stress indicators and eye contact time; the A-not-B task analysis included perseverative error rate and mean time to select a pot, while the detour task included path errors, contact errors, number of direct detours (as dogs could accumulate multiple errors per trial, if detouring unsuccessfully), and time to detour. Component scores were generated using the regression method for each principal component, and these derived values were analysed using linear models to determine statistical differences due to fixed effects of sex, neuter status, age group, breed type, and training experience. The age group and combined training level were included as covariates in this analysis.

All data were analysed using SPSS 27.0 (IBM, New York, NY, USA).

## 3. Results

### 3.1. A-Not-B Task

Dogs were considered successful in the A-not-B task if they selected the ‘B’ pot at least 8/10 times during the test phase. We also separated A-not-B dogs further, splitting them into those that failed the training phase (did not select A correctly five times in a row: FAIL_TRAIN; N = 4), those that passed the training phase, but did not pass the test phase criteria (PASS_TRAIN_FAIL_TEST; N = 15), and those that passed both training and test phases (PASS_TRAIN_PASS_TEST; N = 18). See Supplementary Tables S2 and S3 for the A-not-B data used in the analyses.

**Primary Training:** The primary training discipline had no significant effect on success in meeting the test criteria in the A-not-B task (χ^2^ = 0.960, d.f. = 3, *p* = 0.811), and there was also no significant effect of the primary training discipline on the number of perseverative errors in the A-not-B task (ANOVA: F_3,29_ = 0.497, *p* = 0.688). There was no effect of the primary training discipline (rmANOVA: F_3,27_ = 1.171, *p* = 0.339), trial (rmANOVA: F_3.094,83.539_ = 1.410, *p* = 0.245), or interaction between these factors (rmANOVA: F_9.282,83.593_ = 0.973, *p* = 0.470) on the time to select a pot in the A-not-B task.

**Figure 3:**
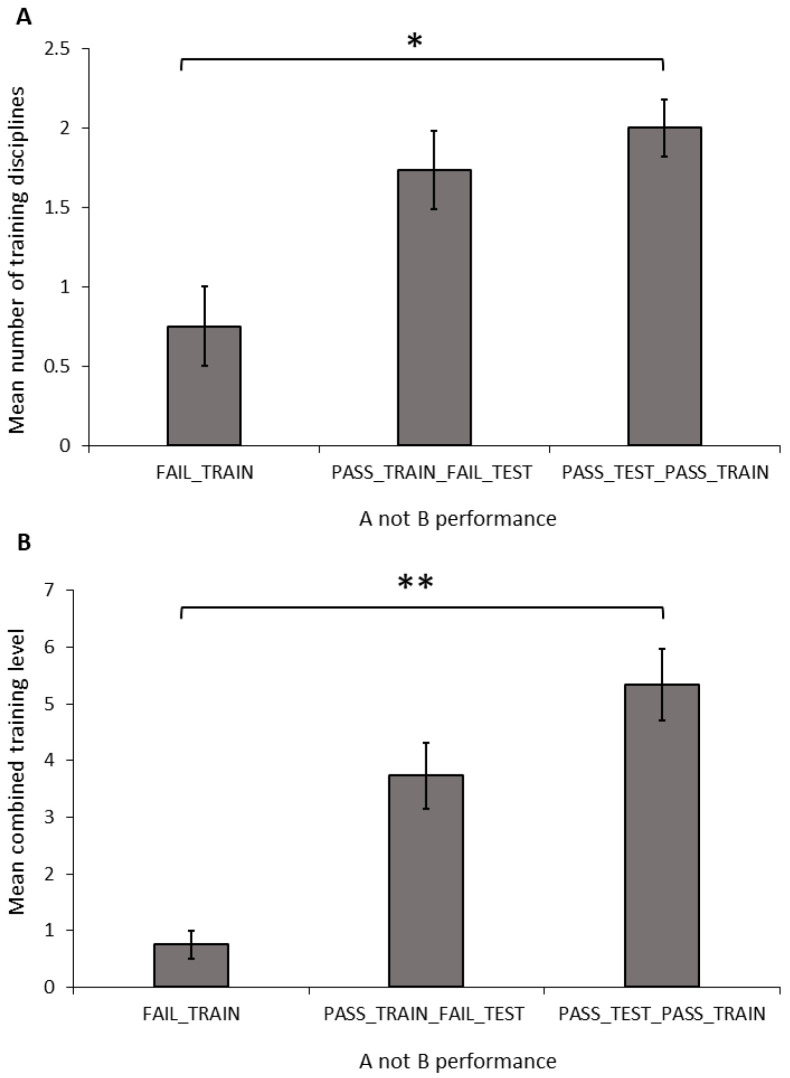
Mean (±SEM) number of disciplines (**A**) and combined training experience (**B**) of dogs grouped by their performance in the A-not-B task. Asterix indicates a significant difference between groups * *p* < 0.05, ** *p* < 0.01.

**Number of disciplines:** The number of training disciplines a dog participated in differed significantly between the performance groups in the A-not-B task (Figure 3A; ANOVA: F_2,34_ = 3.681, *p* = 0.036). The FAIL_TRAIN group took part in significantly fewer disciplines than did the PASS_TRAIN_PASS_TEST group (Sidak: *p* = 0.031); however, there was no difference between dogs in the FAIL_TRAIN and PASS_TRAIN_FAIL_TEST groups (Sidak: *p* = 0.126) or the PASS_TRAIN_FAIL_TEST and PASS_TRAIN_PASS_TEST groups (Sidak: *p* = 0.747). The number of disciplines a dog trained in showed no significant relationship with the number of perseverative errors during the A-not-B task (Pearson’s correlation: r = −0.035, N = 33, *p* = 0.846).

**Combined training experience:** The combined training level differed significantly between the A-not-B groups (Figure 3B; ANOVA: F_2,34_ = 6.428, *p* = 0.004). The FAIL_TRAIN group had a significantly lower combined training experience score compared to that of the PASS_TRAIN_PASS_TEST group (Sidak: *p* = 0.004); whereas the FAIL_TRAIN group did not differ significantly from the PASS_TRAIN_FAIL_TEST group (Sidak: *p* = 0.098); and there was also no significant difference between the PASS_TRAIN_FAIL_TEST group and the PASS_TRAIN_PASS_TEST group (Sidak: *p* = 0.182). The combined training experience showed no significant relationship with the number of perseverative errors during the A-not-B task (Pearson’s correlation: r = −0.227, N = 33, *p* = 0.204).

**Success, speed, and error rate:** Only dogs that completed all ten A-not-B test trials were included in the analyses. Dogs that passed the A-not-B task required fewer trials to select the correct ‘B’ pot (mean ± sem: 1.22 ± 0.10) than dogs that failed the A-not-B task (2.33 ± 0.46; GLM: χ^2^ = 8.392, d.f. = 1, *p* = 0.004). First trial selection predicted whether dogs would go on to meet trial criteria in the A-not-B task (χ^2^ = 4.591, d.f. = 1, *p* = 0.032), with 14/18 dogs that succeeded in reaching the test criteria (8/10) making the correct choice on the first trial, and only 5/13 dogs that failed making the correct choice on the first trial meeting the test criteria.

Dogs did not alter their speed of pot choice significantly across trials in the A-not-B task (rmANOVA: F_2.973,86.217_ = 1.972, *p* = 0.125), nor was there an interaction between success in the task and the speed of choice across the trials (rmANOVA: F_2.973,86.217_ = 1.729, *p* = 0.167); however, dogs that met the criteria (8/10 correct) in the A-not-B task were significantly faster at choosing a pot than those that failed to reach this criteria (rmANOVA: F_1,29_ = 13.949, *p* = 0.001). Dogs that were slower, on average, to choose a pot also made a greater number of perseverative errors overall (Pearson’s correlation: r = 0.528, N = 31, *p* = 0.002).

**Figure 4:**
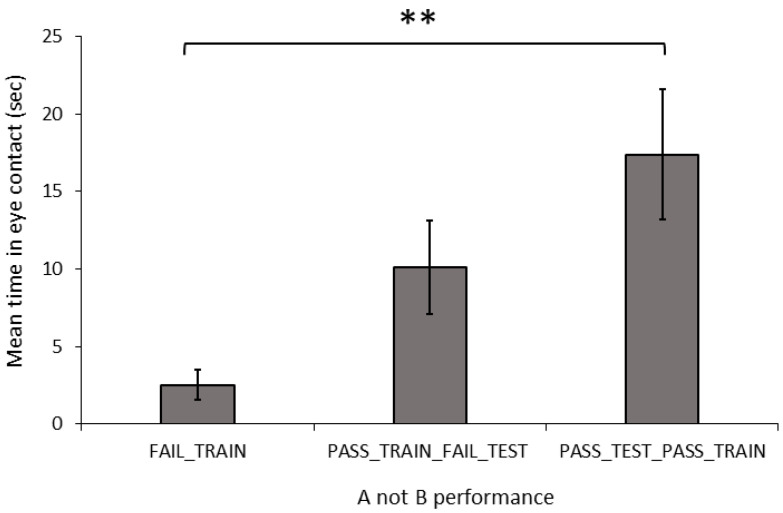
Mean (±SEM) time spent by dogs making eye contact with the owner over 60 s, based on their performance in the A-not-B task. Asterix indicates a significant difference between groups ** *p* < 0.01.

**Behaviour and Dog Characteristics:** In the A-not-B, task there was a significant difference in the duration of eye contact with the owner between the training-testing groups (Figure 4; Welch: F_2,20.467_ = 7.889, *p* = 0.003). The FAIL_TRAIN dogs made significantly less eye contact than did the PASS_TRAIN_PASS_TEST dogs (G-H: *p* = 0.009), whereas the PASS_TRAIN_FAIL_TEST dogs did not differ in the level of eye contact compared to those of the other two groups (FAIL_TRAIN: G-H: *p* = 0.074; PASS_TEST_PASS_TRAIN; G-H: *p* = 0.352). The number of stress indicators did not differ significantly between the three groups (ANOVA: F_2,31_ = 0.067, *p* = 0.936). 

There was no significant relationship between the duration of eye contact with the owner (Pearson’s correlation: r = −0.287, N = 30, *p* = 0.124) or the number of stress indicators (Pearson’s correlation: r = 0.143, N = 30, *p* = 0.452) with the number of perseverative errors across 10 trials in the test for the A-not-B task. 

There was no effect of age group (χ^2^ = 0.027, d.f. = 1, *p* = 0.869), sex (χ^2^ = 0.068, d.f. = 1, *p* = 0.794), neuter status (χ^2^ = 0.160, d.f. = 1, *p* = 0.689), or interaction between neuter status and sex (χ^2^ = 0.002, d.f. = 1, *p* = 0.967), on success in the A-not-B task. Breed category (gundog, pastoral, other) did not significantly affect success in the A-not-B task (χ^2^ = 3.282, d.f. = 2, *p* = 0.194). 

### 3.2. Detour Task

Dogs were considered successful in the detour task if they moved around the barrier in 8/10 trials, without contacting the barrier (contact error) or entering the direct approach zone (path error). A total of 14 dogs met the criteria for the detour task (pass), and 24 did not reach this criteria (fail). See Supplementary Tables S4 and S5 for the detour data used in the analyses.

**Primary Training Discipline:** The primary training discipline did not affect the success rate in the detour task (χ^2^ = 2.934, d.f. = 3, *p* = 0.402). Moreover, the primary training discipline did not impact on the contact error rate (Welch: F_3,11.856_ = 1.860, *p* = 0.191) or the path error rate (ANOVA: F_3,34_ = 0.817, *p* = 0.493), but did significantly affect the total error rate (Welch: F_3,17.247_ = 3.277, *p* = 0.046) in the detour task. However, the results of the Games–Howell post hoc pairwise tests were not significant for any pair-wise comparisons (G-H: *p* > 0.05).

**Figure 5:**
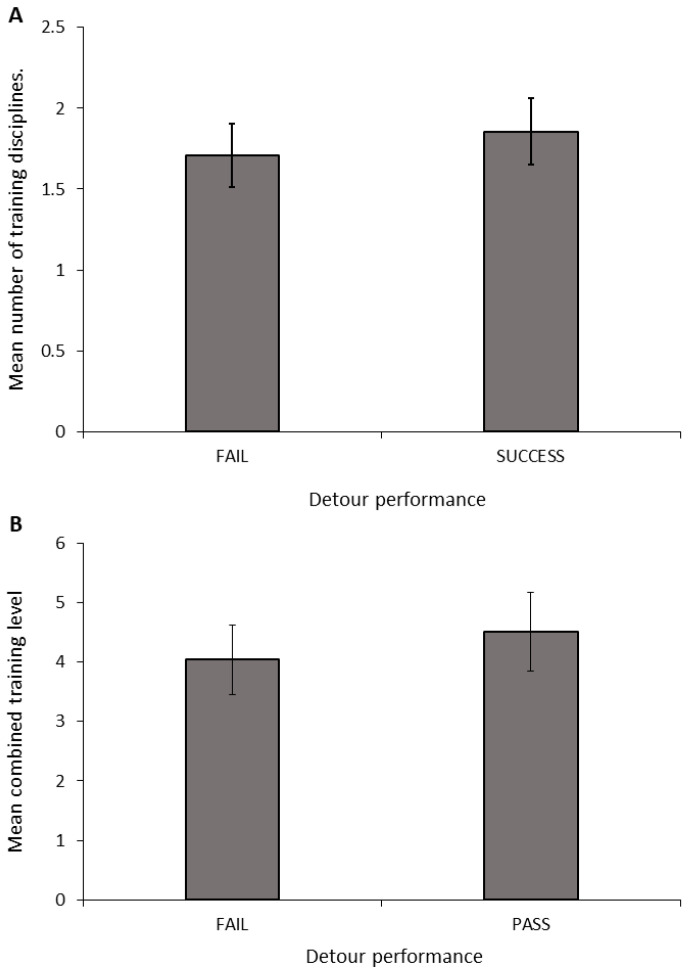
Mean (± SEM) number of disciplines (**A**) and combined training experience (**B**) of dogs grouped by their performance in the detour task.

**Number of disciplines:** The number of disciplines a dog trained in did not differ between dogs that succeeded or failed in the detour task (Figure 5A; ANOVA: F_1,36_ = 0.246, *p* = 0.623). There was also no relationship between the number of training disciplines and the number of contact errors (Pearson’s correlation: r = 0.166, N = 38, *p* = 0.319), the number of path errors (Pearson’s correlation: r = 0.017, N = 38, *p* = 0.918), or the total number of errors (Pearson’s correlation: r = 0.094, N = 38, *p* = 0.575) made during the detour task.

**Combined training experience:** The combined training level across disciplines did not differ between dogs that failed or succeeded in the detour task (Figure 5B; ANOVA: F_1,36_ = 0.249, *p* = 0.621). There was also no relationship between the combined training experience and the number of path errors (Pearson’s correlation: r = −0.083, N = 38, *p* = 0.918) or the total number of errors (Pearson’s correlation: r = 0.142, N = 38, *p* = 0.394) made in the detour task. However, dogs with a greater combined training experience were more likely to contact the barrier in the detour task (Pearson’s correlation: r = 0.422, N = 38, *p* = 0.008).

**Figure 6:**
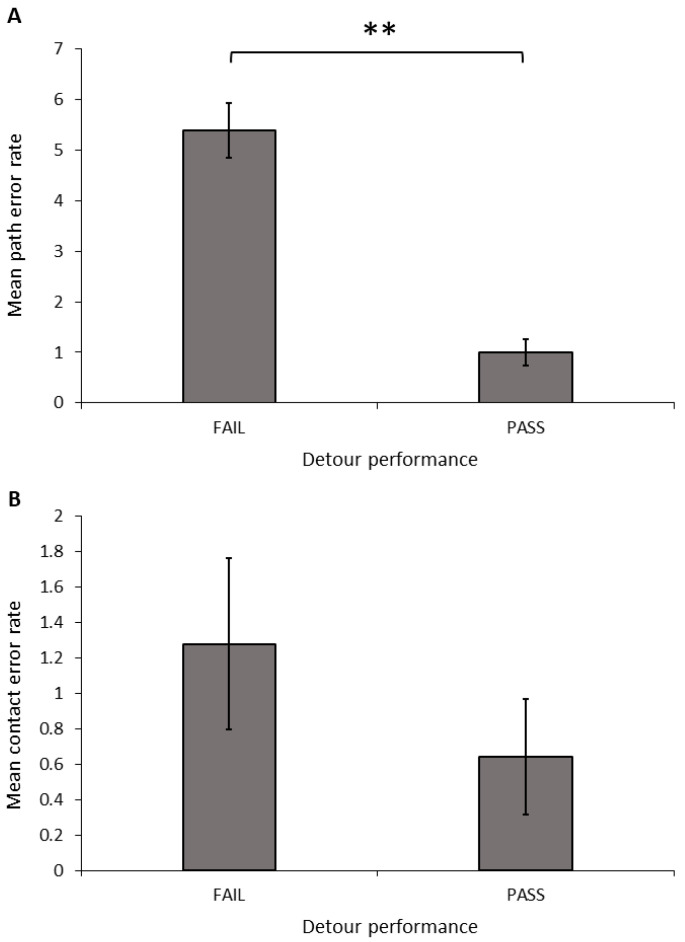
Mean (±SEM) path error rate (**A**) and contact error rate (**B**) across 10 trials, grouped by performance in the detour task. Asterix indicates a significant difference between groups, ** *p* < 0.01.

**Success, speed, and error rate:** Only dogs that completed all ten detour test trials were included in the analyses. Dogs that succeeded in meeting the criteria in the detour task made significantly fewer path errors (Figure 6A; *t*-test equal variances not assumed: *t* = 6.670, d.f. = 23.887, *p* < 0.001) and total errors (*t*-test equal variances not assumed: *t* = 5.568, d.f. = 24.447, *p* < 0.001) than dogs that failed to reach these criteria; however, there was no significant difference in the number of contact errors (Figure 6B; *t*-test: *t* = 1.024, d.f. = 30, *p* = 0.314). We also found a significant relationship between the successful detour rate and both the path error rate (Spearman’s correlation: r = −0.997, N = 32, *p* < 0.001) and the total error rate (Spearman’s correlation: r = −0.943, N = 32, *p* < 0.001). Dogs with a higher detour success rate had a lower number of path errors and total errors, but there was no relationship between the success rate and the contact error rate (Spearman’s correlation: r = 0.204, N = 32, *p* = 0.263).

The dogs did not alter their detour speed significantly across the trials (rmANOVA: F_3.609,108.283_ = 1.545, *p* = 0.132), nor was there an interaction between success in the task and the speed of detour (rmANOVA: F_3.609,108.283_ = 0.959, *p* = 0.474); however, dogs that met the criteria (8/10 correct) in the detour task were significantly faster (3.143 s, CI: 1.477, 4.810) in moving around the barrier than those that failed to reach the criteria (7.442 s, CI 5.973, 8.912; rmANOVA: F_1,30_ = 15.615, *p* < 0.001). 

The dogs that took more time on average to complete the detour task, on average, made a greater number of path errors (Pearson’s correlation: r = 0.518, N = 32, *p* = 0.002) and total errors (Pearson’s correlation: r = 0.370, N = 32, *p* = 0.037), but there was no relationship between the average detour time and the contact errors (Pearson’s correlation: r = −0.062, N = 32, *p* = 0.738). 

**Behaviour and dog characteristics:** There was no significant difference in the number of stress indicators (ANOVA: F_1,33_ = 1.204, *p* = 0.281) or time spent watching the owner (ANOVA: F_1,33_ = 1.810, *p* = 0.188) between the dogs that passed or failed the detour task. 

We found no significant relationship between error rates and the duration of eye contact with the owner (Pearson’s correlation: contact error rate: r = 0.091, N = 35, *p* = 0.603; path error rate: r = −0.232, N = 35, *p* = 0.181; combined error rate: r = −0.164, N = 35, *p* = 0.346). There was a significant relationship between the number of stress indicators and the number of path errors that dogs made during the detour task (Pearson’s correlation: r = −0.336, N = 35, *p* = 0.048); dogs that exhibited more stress indicator signals made fewer path errors across the 10 detour trials. However, the contact error rate (Pearson’s correlation: r = −0.044, N = 35, *p* = 0.603) and the total error rate (Pearson’s correlation: r = −0.164, N = 35, *p* = 0.346) showed no relationship with the number of stress indicators.

The age group did have a significant effect on success in the detour task (χ^2^ = 4.284, d.f. = 1, *p* = 0.038), with younger dogs performing better than older dogs, but there was no effect of sex (χ^2^ = 0.803, d.f. = 1, *p* = 0.370), neuter status (χ^2^ = 1.672, d.f. = 1, *p* = 0.196), or interaction between neuter status and sex (χ^2^ = 0.827, d.f. = 1, *p* = 0.363) on success in the detour task. Breed category had no effect on the success rates in the detour task (χ^2^ = 1.433, d.f. = 2, *p* = 0.488). 

### 3.3. Comparison of Performance in A-Not-B and Detour Tasks

**Figure 7:**
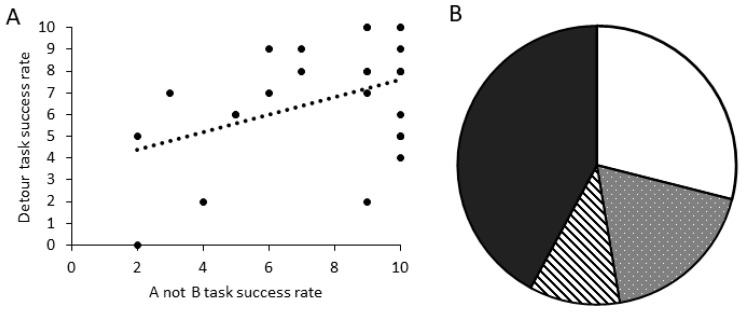
Performance across tasks showing: (**A**) the relationship between successful trials in the A-not-B task and the detour task for dogs that completed 10 trials in both tasks (N = 27); and (**B**) the proportion of all dogs tested that met the task criteria (8/10 success) in both tasks (white; N = 10), in the A-not-B only (spotted; N = 8), in the detour only (striped; N = 4), or in neither task (black; N = 16) (N = 38).

There was a significant positive relationship between the number of correct trials in the A-not-B task and the detour task for dogs that completed all 10 trials in both tasks (Figure 7A; GLM score comparison: χ^2^ = 5.194, d.f. = 1, *p* = 0.023). When comparing criteria performance, dogs that were successful in the A-not-B task (PASS_TRAIN_PASS_TEST) were significantly more likely to also be successful in the detour task (Figure 7B; GLM binomial pass/fail: χ^2^ = 5.355, d.f. = 1, *p* = 0.021). 

There was no significant relationship between the number of perseverative errors dogs made in the A-not-B task and errors made during the detour task in regards to the contact error rate (Pearson’s correlation: r = −0.147, N = 33, *p* = 0.414), the total error rate (Pearson’s correlation: r = 0.155, N = 33, *p* = 0.388), or the path error rate (Pearson’s correlation: r = 0.325, N = 33, *p* = 0.065).

### 3.4. Principal Components Analysis

Overall, the PCA revealed three underlying behavioural components, which explained 72.3% of the cumulative variation in behaviour (Table 3). PC1 included speed and path errors in the detour task, as well as time to select a pot and perseverative errors in the A-not-B task. A high PC1 score represents dogs that took longer to perform both tasks and committed a greater number of errors in both tasks. We considered this to be a good measure of inhibitory control, those dogs with a high score in PC1 showed low inhibitory control. PC2 included stress indicators score and time spent in eye contact with the owner. A high PC2 score covaried with an increased number of stress indicators and increased duration of eye contact, suggesting that this component was related to attachment and arousal behaviour, with anxious dogs seeking owner reassurance in uncertainty. PC3 included contact errors only; a high PC3 score represented dogs that made a high number of contact errors. Contact errors were not related to task success or other errors (see above), but from observation, appeared to represent the dogs attempting an alternative solution to the task; therefore, we consider this to be a measure of persistence. Dogs with a low PC3 score are more likely to persist in repeating the previously rewarded behaviour, whereas dogs with a high PC3 score are more likely to try alternative ways to access the reward.

**Figure 8:**
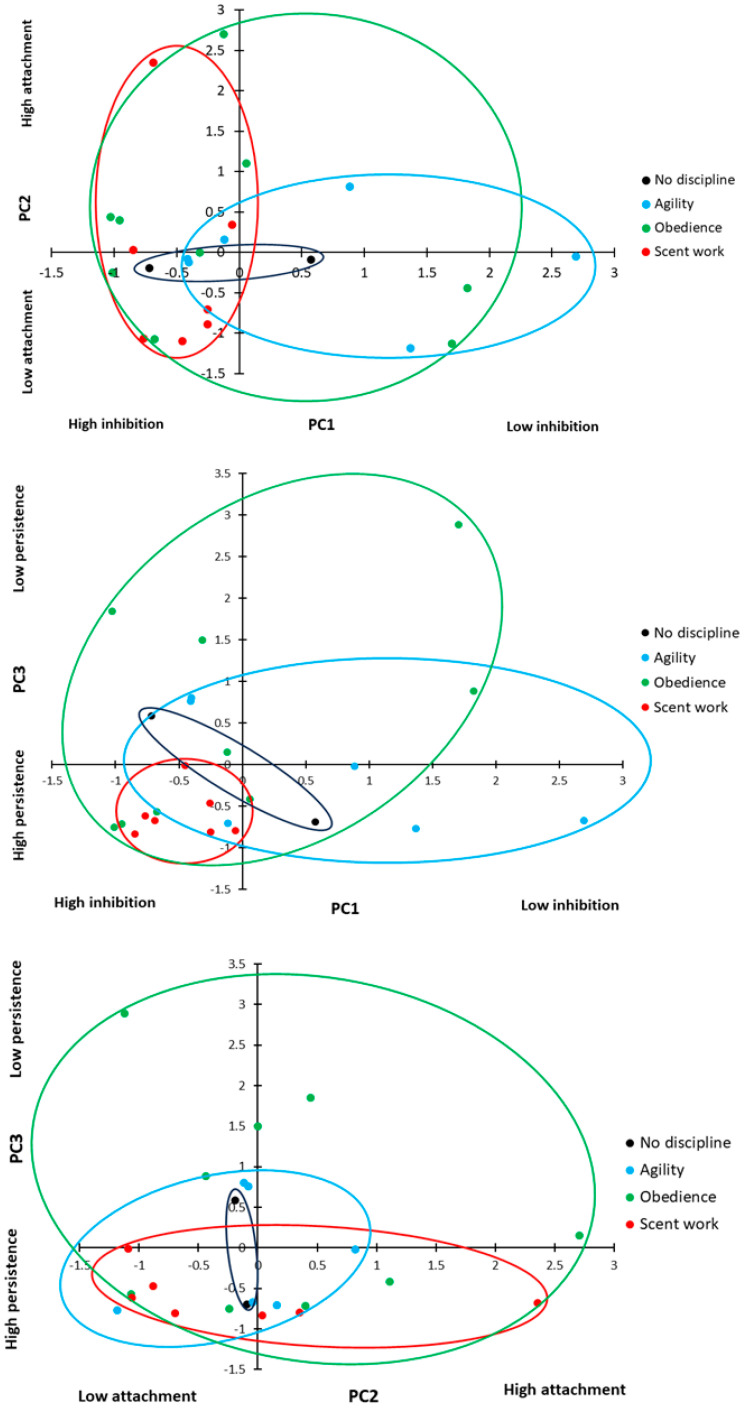
Principal components displayed by primary training discipline. PC1 = inhibition (inhibitory control); PC2 = attachment (and arousal); PC3 = persistence.

There was no difference in inhibitory control (PC1: inhibition) scores between dogs based on the discipline number (ANOVA: F_2,17_ = 0.514, *p* = 0.607) or a relationship between combined training experience and inhibitory control (ANOVA: F_1,17_ = 2.075, *p* = 0.136). However, there was a significant difference in inhibitory control based on the primary training discipline (Figure 8; ANOVA: F_2,17_ = 3.467, *p* = 0.035). Dogs trained primarily in scent work showed significantly higher levels of inhibitory control compared to dogs trained primarily in agility (Figure 8; Sidak: *p* = 0.012), but there was no difference between the other primary disciplines (Sidak: *p* > 0.05 for all comparisons). There was no significant effect of sex, neuter status, breed group, or age group on inhibitory control (ANOVA: sex: F_1,18_ = 0.603, *p* = 0.447; neuter status: F_1,18_ = 0.487, *p* = 0.494; breed group: F_2,18_ = 0.732, *p* = 0.495; age group: F_1,18_ = 2.001, *p* = 0.174). 

Attachment and arousal behaviour (PC2: attachment) scores showed no difference between the primary training disciplines (Figure 8; ANOVA: F_2,17_ = 0.754, *p* = 0.485) or the number of disciplines a dog trained in (ANOVA: F_2,17_ = 0.220, *p* = 0.805), and there was no relationship with overall training experience (ANOVA: F_1,17_ = 2.447, *p* = 0.136). There was no significant effect of sex, neuter status, breed group, or age group on attachment (ANOVA: sex: F_1,18_ = 0.054, *p* = 0.819; neuter status: F_1,18_ = 0.940, *p* = 0.345; breed group: F_2,18_ = 0.275, *p* = 0.763; age group: F_1,18_ = 0.787, *p* = 0.387). 

There was a significant difference in persistence (PC3: persistence) scores between dogs with different primary disciplines (Figure 8; GLM: χ^2^ = 7.029, d.f. = 2, *p* = 0.030); dogs trained in scent work showed higher persistence (a lower contact rate) than dogs trained in obedience (*t* = 2.519, d.f. = 8.984, *p* = 0.033); however, there was no significant difference between the other training groups (agility vs. scent work: *t* = 1.559, d.f. = 6.318, *p* = 0.167; agility vs. obedience: t = 1.200, d.f. = 12.780, *p* = 0.252). There was no significant effect of sex, neuter status, breed group, or age group on persistence (ANOVA: sex: F_1,18_ = 0.447, *p* = 0.512; neuter status: F_1,18_ = 0.628, *p* = 0.438; breed group: F_2,18_ = 0.350, *p* = 0.710; age group: F_1,18_ = 0.057, *p* = 0.815). 

## 4. Discussion

Previous studies on the impact of training on dog cognition and inhibitory control have largely partitioned dogs into ‘highly trained’ (work or sports) vs. untrained pet dogs, without considering whether the type of training a dog undertakes influences its cognitive ability [18,23]. The main goal of our study was to determine whether training in a specific discipline enhances the cognitive ability of pet dogs in tasks that require inhibitory control (A-not-B and detour task), comparing scent work, agility, and obedience. We predicted *a priori* that dogs trained primarily in scent work would outperform dogs trained primarily in agility or obedience due to the requirements for dogs trained in scent work to develop both handler independence [24] and inhibitory control [25,26]. We found no evidence to support this hypothesis in the individual tasks, with primary training discipline failing to predict success or error rate in either the A-not-B task or the detour task. However, the primary training discipline did affect dog behaviour across the tasks. Scent dogs exhibited higher overall inhibitory control compared to agility dogs, as well as greater persistence in repeating the rewarded behaviour in the detour task compared to the behaviour of the obedience trained dogs. These results are similar to those of Carballo et al. [18], who compared the performance of assistance/support dogs to trained family dogs (primarily obedience and agility trained) or untrained family dogs. Scent work requires dogs to perform one task across multiple contexts (find the scent and indicate or track), whereas in agility and obedience training, dogs must perform multiple different behaviours, thus relying on human direction to know which behaviour is expected of them. Assistance dogs, like scent work dogs, require a high level of independence and persistence, alongside inhibitory control, to avoid distractions and perform successfully [44]. While the cognitive task was different, involving the use of a puzzle box rather than the A-not-B or detour tasks, the assistance dogs outperformed the trained and untrained family dogs, demonstrating less social dependence [18]. Scent work training has also been shown to create a positive bias in dogs [45]; however, we found no difference between scent dogs and dogs trained in other disciplines to support an effect of the training discipline on attachment and arousal levels, indicating that differences in arousal levels between disciplines in our cohort was not the main factor impacting on overall performance. 

In line with previous studies [6], we found that combined training experience affected success in the A-not-B task; dogs that succeeded in the A-not-B (at least 8/10 correct) task had been trained in more disciplines and to a higher combined training level compared to dogs that failed to pass the initial training phase. The lower performance may be due to lack of attention to experimenter; dogs that failed the training stage also made less initial eye contact with their owner, indicating they may have paid less attention to the human-directed indication of the correct pot to select to obtain food during the ‘A’ training trials. Despite previous training experience impacting on the initial training phase, we found no significant effect of training level on either criteria or perseverative search errors in dogs that had passed the training phase and no relationship between training experience and the number of successful trials. Attention to human signals is thought to play an important role in success in the A-not-B task, but previous studies have primarily shown that ostensive human communication (e.g., physical gestures such as pointing or gaze shift) after placing the ‘B’ reward leads to errors rather than a positive effect on performance [35]. There was no significant relationship between performance in the test element for dogs that passed the training stage and prior training experience, contrary to the previous study, which found that highly trained water rescue dogs made fewer perseverative errors during the ‘B’ test phase compared to untrained dogs [6]. Our sample size (N = 37) was lower than that used in this previous study (N = 48), perhaps reducing our ability to detect an effect of training. In addition, in our study, combined training experience was considered on a linear scale, rather than partitioning dogs between highly trained and untrained, covering a wider range of experience. 

Irrespective of training experience, dogs that took longer to make a pot selection in the A-not-B task during the test phase were also more likely to make perseverative errors and fail to meet our criteria. The task may have been more challenging for dogs that have greater reliance on human direction; those taking longer to select a pot may have been waiting for their handler to provide the correct indication [27]. However, our data showed that dogs who make more eye contact with their handler performed better on the A-not-B task, indicating that this is unlikely. The relationship between latency and performance may instead have been due to motivation for the reward [45]. Very high-value rewards can decrease inhibitory control through increased arousal, negatively impacting on cognitive performance that requires inhibition [46]. A longer latency between release and pot selection may suggest that these dogs were unfocused and unable to inhibit their distracted behaviour, therefore taking longer to initially select a pot. Alternatively, perceived high-value rewards can also increase the speed for obtaining the reward [47] and the attention to human communication in placement of the reward [48]. Therefore, relative perceived reward value could either hinder or improve performance. We did not determine how valuable the reward was to the dogs, only that they would perform the action of pot selection to obtain it during the familiarisation trials. Perceived reward value and how this impacts on performance has not been tested in the A-not-B task, but it does impact on performance in the cylinder task [46] and inhibitory control in social interaction tasks [20]; therefore, it should be considered in future work. 

Previous studies have also found enhanced performance on the detour task in highly trained dogs [7,16]; however, we found no evidence to support that increased training experience improves performance in pet dogs. This may have been due to a lack of variation in the arousal level related to training experience among the dogs that took part in the test; arousal level has been found to strongly influence detour task performance [7]. However, dogs with higher combined training experience were more likely to contact the barrier. Barrier contact rate was not associated with the overall level of performance, either in terms of detour speed or ability to meet our detour task criteria (8/10 direct), nor was it related to the frequency of path errors, which was higher in dogs that failed the detour task. This contrasts with previous research in which barrier contact was a good indicator of detour performance [7]. Given that highly trained dogs outperform those with less training on detour tasks and also exhibit increased inhibitory control to directly approach the reward [7,8], the positive relationship between contact errors and training experience seems to be counterintuitive. From our observations, it appeared that contact errors were more strongly associated with dogs attempting an alternative solution to the detour behaviour to gain access to the reward, rather than simply a lack of inhibition resulting in a direct approach to the food. Highly trained dogs have been shown to demonstrate increased puzzle solving abilities [15,17,18]; therefore, it could be that those dogs with a high barrier contact rate were trying to ‘solve’ the puzzle of how to gain the reward without performing the detour behaviour. A direct comparison of puzzle solving ability and detour behaviour would be required to determine if this might be the case. 

While contact error rate did not predict detour success, path error rate, in which the dog initially directly approaches the reward, rather than uses the shortest route to detour the barrier, did relate to success. A higher path error rate was strongly associated with low direct detour success across the ten trials, as found in previous work [7]. Dogs with lower path error rates also detoured the apparatus faster [7,49], demonstrating that inhibitory control of the direct approach to the reward improved speed of performance as well as accuracy. Whilst previous training experience did not alter path error rate or overall success, dogs with a higher number of stress indicators were less likely to make path errors during the detour task. In this task the experimenter holding the food bowl is directly facing the dog, a reduction in path errors may be due to a reluctance in dogs that have higher stress levels to directly approach a stranger [50]. Alternatively, stress can alter the way rewards are perceived [51], and higher stress or arousal levels may devalue the reward increasing inhibition of direct approach behaviour in the detour task. 

Irrespective of training experience, dogs that succeeded in the A-not-B task were more likely to also succeed in the detour task, potentially as both tasks require inhibitory control [7,35]. This result is contrary to previous studies that have found no relationship in performance between the A-not-B task and the cylinder task or detour task [20,43,52,53] and lack of general support in the literature for correlations in performance across tasks requiring impulse control is confirmed in a recent review [54]. Whilst the detour task is considered comparable to the cylinder task [7], the difference in motor control, reaching vs. whole body movement around a barrier, and requirement in the detour task to initially move away from the food may lead to important differences in the executive functions required. In a comparison between wolves and dogs on a detour vs. cylinder task, wolves outperformed dogs in the detour task, whereas dogs were more efficient in the cylinder task [46] indicating differences in the cognitive function tested using these two tasks. In the only other study to date using both A-not-B and a detour task, Vernouillet et al. [52] tested the number of trials it took for the dog to successfully choose location ‘B’ in the A-not-B task, but did not continue to test the dog’s performance beyond the first successful choice. We found 8/18 dogs that chose the ‘B’ location correctly the first time during the test trials went on to make perseverative errors in subsequent trials, and 14/18 dogs that selected the ‘B’ pot correctly in the first test trial passed our criteria across the 10 trials. Therefore, while dogs that were successful on the first trial were more likely to reach our test criteria, the use of the first trial only did not always predict success, and this result may fail to capture some of the variation in behaviour among individuals. 

An issue raised in the comparison of performance across multiple tasks is the ‘ceiling response’, which limits the range of cognitive performance between dogs and thus greatly restricts the ability to find a significant relationship between performance in different cognitive tasks [20,23,54]. In this respect, our success rate, 14/38 (37%) for the detour task, was comparable to that of 45% obtained in a similar study [16]. However, for the A-not-B task, our success rate 18/37 (48%) was considerably lower than the rate obtained previously (e.g., >80%) in other studies [20,52]. The latter, potentially reflects our higher criteria level to pass the training phase for this task, requiring five successive correct choices measured across ten trials for all dogs, irrespective of performance. The dogs in our study therefore had greater fidelity to the ‘A’ pot due to additional training trials. Running multiple test trials in each task would have enhanced the accuracy of the measurements of dog cognitive performance, and therefore not only increased variation within task, but also increased the ability to identify a relationship in performance between tasks. This was confirmed by the PCA analysis where several measurements of inhibitory control across both tasks (error rate, task completion time), loaded onto a single principal component that explained 37% of the behavioural variation among dogs. Furthermore, the sample size in our study (N = 37) was higher than the sample sizes in other studies that compared performance in the A-not-B task with the cylinder or detour task (N = 11 to 34:54). Therefore, the lower sample sizes across these studies, along with variation in the way in which successes and errors in tasks were determined, may have led to a mismatch between our results and those of previous researchers [23,52,54]. 

Despite a relationship between success in the A-not-B and the detour tasks, showing a common component of cognitive ability, we found no relationship between the error rates across the two tasks. During the detour task, in addition to inhibiting a direct approach to the food, perseveration in the direct path is rewarded, whereas in the A-not-B task, perseveration in choosing the A pot is not rewarded, so the A-not-B task requires a greater degree of cognitive flexibility, in addition to inhibitory control [52]. Errors in the A-not-B task represent a lack of cognitive flexibility to inhibit the previously rewarded response, whereas errors in the detour task are due to a lack of inhibition to approach the food directly, but may also include aspects of spatial perception and navigation to successfully detour the barrier [23,54]. Therefore, a comparison of errors rather than of successes may be inappropriate when comparing the performance between these tasks. 

Stress indicators upon first entering the test environment did not provide a good predictor of cognitive performance, despite predictions that levels of arousal would significantly impact on cognitive ability [7,32]. There was a positive relationship in the PCA analysis between eye contact and stress indicators, although PC2 did not predict cognitive performance. Increased eye contact has been found to indicate a stronger attachment between the dog and owner [38], and the positive relationship between stress indicators and eye contact duration suggests that dogs experiencing higher stress levels were also seeking owner reassurance [55]. Owner presence enhances problem solving ability in dogs, irrespective of the owner’s behaviour [56]. The lack of a relationship between stress indicators and general cognitive performance may therefore have been due to owners remaining present throughout the tasks, reducing the impact of initial stress levels on cognitive performance.

Previous studies have found effects of both breed type [16] and sex [30] on cognitive performance, but we found no support for this in either task. Breed tends to influence the level of attention paid to human signals [10,11,12,13]. This is considered important in the A-not-B task, where success is related to attention to the experimenter positioning the reward [57], but not in the detour task, when the behaviour is not demonstrated. Experience has also been found to reduce breed differences in cognitive traits [13,14], and so a variable training background across our participants may have reduced the likelihood of finding breed-related differences in behaviour. Neuter status had no effect on performance in either task, confirming previous work indicating that it has no effect on cognitive tasks [30]. We found no effect of age group on performance in the A-not-B task, but age affected performance in the detour task. Younger dogs were more likely to meet the detour criteria than were older dogs, potentially due to a general decline in cognitive function with age [58]; however, age appeared not to impact on performance in other detour studies [7,11,16]. A lack of effect of age in the A-not-B task indicates that this response is less likely to be due to cognitive decline in general affecting inhibitory control, but may potentially be due to differences in navigation and spatial perception. For example, allocentric navigation (use of landmarks to navigate a route) shows a significant decline with age in dogs [59], and exploratory behaviours are higher in younger dogs [60]. This may lead to a greater tendency in younger dogs to navigate successfully around the barrier and remember their route on subsequent trials. 

While this study clearly points towards an effect of prior training discipline on inhibitory control in pet dogs, the results do not show strong support for training enhancing cognitive performance. This may be due to limitations in the current study. For example, a relatively small sample size in comparison to previous studies may have reduced the ability to detect the effects of sex or breed type [16,30]. Additionally, the study relied on owner identification of the breed of participants; however, breed identification by visual characteristics can be unreliable [61]. The incorrect allocation of dogs to specific breed groups may have confounded the results, and the verification of breed, either by access to registration details or DNA analysis, may be beneficial in future studies. Testing dog performance on only two tasks may also have reduced our ability to detect the impact of training experience. Previous studies comparing highly trained dogs to untrained dogs have used a greater range of cognitive tasks, but these only detected an impact of training on some of the tasks used [23,54]. Therefore, further work is needed to determine whether pet dog training experience can impact on performance across a wider range of cognitive tasks.

## 5. Conclusions

Pet dog performance in the A-not-B task and detour task was related, with dogs that performed better on one task also being more likely to perform better on the other. This indicates an underlying cognitive trait that enhances performance across both tasks, potentially inhibitory control. Training discipline and experience did not affect performance in the individual tasks; however, dogs that trained primarily in scent work demonstrated stronger inhibitory control across tasks and more persistence in the detour task than did the agility dogs or obedience dogs, respectively. This suggests that scent-work training can have a positive effect on these traits, irrespective of the breed or sex of the dog [24]. However, our data also show that the combined level of training in pet dogs may not have a strong influence on performance in the A-not-B task or detour task, contrary to the results of studies contrasting untrained pet dogs with highly trained working or sporting dogs [6,16]. The results show that while pet dog training does not influence cognitive problem solving to the same degree as that found in highly trained dogs, our data support the principal that future cognitive studies should consider the training discipline [18], rather than grouping ‘highly trained’ dogs across different disciplines into a single category. 

## Figures and Tables

**Table 1:** Training class levels within each discipline and corresponding training level score. The combined training level was calculated by combining the training level score from an individual’s primary discipline with the training level score obtained in other disciplines if the individual engages in multiple disciplines. Dogs received a score of ‘0’ if they did not participate in training.

Training Level Score	Obedience Training Class Levels	Scent-Work Training Class Levels	Agility Training Class Levels
1	Introductory—heelwork (lead only), recall, and use of a toy/food as reward.	Level one—Dog to recognise one scent (cloves) on one article, in three out of four search areas, with 3 min. per search area.	Beginner—Can sequence some obstacles, but not currently completing all obstacles at full height.
2	Pre-Beginner—heelwork on lead, heelwork free, and recall.	Level two—Dog to recognise one scent (cloves) on two articles, in one perimeter and one exterior (item hidden) search, with 5 min. per search area. Man trailing level 1: Single blind, trail length 200–400 m, trail age 30–60 min.	Pre-Competition—Can complete all obstacles, but has not competed in any events. Jumps may not be at full height.
3	Beginner—heelwork on lead, heelwork free, recall, and retrieve any article.	Level three—Dog to recognise two scents (cloves and gun oil), in one perimeter and one exterior (item hidden) search, with 5 min. per search area.	Competition—Has competed in independent shows with all obstacles at full height, but has not participated in KC or UKA competition.
4	Novice—heelwork on lead, heelwork free, recall, and retrieve a dumbbell.	Level four—Dog to recognise two scents (cloves and gun oil) with two distraction scents, in one perimeter and one exterior (item hidden) search, with 5 min. per search area. Man trailing level 2: Double blind, trail length 400–600 m, trail age 1–2 h.	Competition grade 1–3—Competing at KC Grade 1–3 or UKA beginner.
5	Class level A—heelwork free, recall, retrieve a dumbbell, handler scent discrimination.	Level five—Dog to recognise two scents (cloves and gun oil) and find 9 out of 12 possible scented articles in both interior and exterior searches.	Competition grade 4–5—Competing at KC Grade 4–5 or UKA novice.
6	Class level B—heelwork free (fast and slow), send away and recall, retrieve one article supplied by a judge, handler scent discrimination with decoy scent.	Level six—Dog to recognise three scents (cloves, gun oil, truffle oil) and find 9 out of 12 possible scented articles in both interior and exterior searches. Man trailing level 3: Double blind, trail length 600–1000 m, trail age 2–4 h.	Competition grade 6–7—Competing at KC Grade 6–7 or UKA senior.
7	Class level C—heelwork free (fast and slow), send away and recall, retrieve one article supplied by the judge, distance control (sit, stand, down), judge scent discrimination with one or more decoy scents.	Level seven—Dog to recognise three scents (cloves, gun oil, truffle oil) and find 9 out of 12 possible scented articles in both interior and exterior searches. Each search area to include a decoy, unscented, article.	Competition level—Competing at KC championship or UKA championship.

**Table 2:** Descriptive characteristics for canine subjects (*n* = 40). M = intact male, F = intact female, M * = neutered male, F * = neutered female. See data in Supplementary Material Table S1 for full breed descriptor.

Subject ID	Sex	Age Group	Breed Type	Primary Training Discipline
1	M *	>8 years	Gundog	Obedience
2	F	2–4 years	Gundog	Scent work
3	F	6–8 years	Gundog	Agility
4	M *	6–8 years	Pastoral	Agility
5	F *	6–8 years	Other	Agility
6	M	2–4 years	Pastoral	Obedience
7	F	<12 months	Pastoral	None
8	M *	1–2 years	Pastoral	Agility
9	F *	2–4 years	Other	Agility
10	M	4–6 years	Other	Obedience
11	F	2–4 years	Other	Obedience
12	M *	>8 years	Other	Obedience
13	M *	2–4 years	Gundog	None
14	F	1–2 years	Other	None
15	F	2–4 years	Other	Scent work
16	F	<12 months	Pastoral	Obedience
17	M	<12 months	Pastoral	Obedience
18	M	2–4 years	Pastoral	Obedience
19	F *	>8 years	Pastoral	Agility
20	F *	>8 years	Other	Scent work
21	F *	>8 years	Pastoral	Obedience
22	F *	>8 years	Pastoral	None
23	F *	1–2 years	Pastoral	Scent work
24	M	2–4 years	Gundog	Scent work
25	F	6–8 years	Gundog	Scent work
26	F	2–4 years	Gundog	Scent work
27	F *	2–4 years	Pastoral	Obedience
28	M *	4–6 years	Gundog	Agility
29	F *	6–8 years	Gundog	Obedience
30	M *	2–4 years	Pastoral	Obedience
31	F	1–2 years	Gundog	Obedience
32	F *	2–4 years	Pastoral	Obedience
33	F *	2–4 years	Other	Obedience
34	M *	2–4 years	Pastoral	Obedience
35	F	<12 months	Pastoral	Scent work
36	F *	2–4 years	Pastoral	Scent work
37	F	2–4 years	Pastoral	Agility
38	M	2–4 years	Gundog	Scent work
39	F *	1–2 years	Gundog	Scent work
40	F	2–4 years	Pastoral	Obedience

**Table 3:** PCA analysis results based on behaviour across the trails. Kaiser–Meyer–Olkin measure of sampling adequacy = 0.535; Bartlett’s test of sphericity: χ^2^ = 40.843, d.f. = 21, *p* = 0.006.

Variable	PC1: Inhibition	PC2: Attachment (and Arousal)	PC3: Persistence
A-not-B perseverative errors	0.877		
Detour time	0.856		
A-not-B pot selection time	0.711		
Detour path errors	0.622		
Stress indicators		0.881	
Eye contact time		0.685	
Detour contact errors			0.953
% Variance	36.846	19.336	16.155
Cronbach’s alpha	0.754	0.331	N/A

## Data Availability

Data are available as Supplementary Material.

## Supplementary Materials

The following supporting information can be downloaded at: https://www.mdpi.com/article/10.3390/ani14030428/s1. Supplementary Materials document including: Abbreviated survey details; Data used for analysis including: Table S1: Background Data, training experience, eye contact duration and number of stress signals; Table S2: A not B trial data indicating success rate at each stage for the A not B trials; Table S3: A not B trial data including number of correct choices, preservative error rate and speed to select a cup (sec) for dogs that completed all 10 TEST trials; Table S4: Detour task showing success rate, number of trials completed and first trial on which dogs successfully detoured without error; Table S5: Detour task trial data only including dogs that completed all 10 detour task trials, including direct detour rate, error rate and detour time (sec).
