# Supplementary material for: Impact of Training Discipline and Experience on Inhibitory Control and Cognitive Performance in Pet Dogs
Source: Animals (Basel). 2024 Jan 29;14(3):428. doi: 10.3390/ani14030428 (PMC10854632; doi:10.3390/ani14030428)
Supplement: Supplementary file 1 [file animals-14-00428-s001.zip › animals-2814215-supplementary.pdf]

Supplementary material for 'Impact of Training Discipline and Experience on Inhibitory Control and Cognitive Performance in Pet Dogs'

Authors: Nerys Mellor, Sebastian McBride, Emma Stoker and Sarah Dalesman.

Abbreviated Survey details:

We have removed questions related to confirmation that owners/handlers are over 18 and agree to their data being anonymised and used for the purposes of publication. We have also removed questions we used to gather participant contact details, first name, phone number and e-mail address, and their availability to take part in the cognitive trials. We confirmed whether dogs or handlers had any allergies, and if the dog had any history of reactivity towards other dogs. This was used to schedule dogs so there was no possibility of overlap in time with other participants. We also conformed if the dog had any aggression towards humans, which excluded them from the study.
Background information on dogs gathered via survey and used for analysis (* = must answer):

What sex is your dog? *

male

female

What is the neuter status of your dog? *

Neutered

Entire

How old is your dog? *

< 12 months

1 - 2 years

2 - 4 years

4 - 6 years

6 - 8 years

> 8 years

What breed is your dog? *

Free text response

Does your dog have any formal background in obedience training? If yes, please proceed to the next question. If no, please skip the next question. *

Yes

No

What level of obedience training has your dog achieved?

Introductory class level - heelwork (lead only), recall, and use of a toy/food as reward.

Pre-Beginner class level - heelwork on lead, heelwork free, and recall.

Beginner class level - heelwork on lead, heelwork free, recall, and retrieve any article.

Novice class level - heelwork on lead, heelwork free, recall and retrieve a dumbbell.

Class A level - Heelwork free, recall, retrieve a dumbbell, handler scent discrimination.

Class B level - Heelwork free (fast and slow), send away and recall, retrieve one article supplied by a judge, handler scent discrimination with decoy scent.

Class C level - Heelwork free (fast and slow), send away and recall, retrieve one article supplied by the judge, distance control (sit, stand, down), judge scent discrimination with one or more decoy scent.

Does your dog have any formal background in scent-work training? If yes, please proceed to the next questions - scent-work levels are provided for both ScentworkUK and for MantrailingUK, please select the relevant criteria for your dog whether that be one or both. If no, please skip the next questions.*

Yes

No

What level of scent-work has your dog achieved? (equivalent to ScentworkUk criteria levels).

Level 1 - Dog to recognise one scent (cloves) on one article, in three out of four search areas with 3 minutes per search area.

Level 2 - Dog to recognise one scent (cloves) on two articles, in one perimeter and one exterior (item hidden) search, with five minutes per search area.

Level 3 - Dog to recognise two scents (cloves and gun oil), in one perimeter and one exterior (item hidden) search, with five minutes per search area.

Level 4 - Dog to recognise two scents (cloves and gun oil) with 2 distraction scents, in one perimeter and one exterior (item hidden) search, with five minutes per search area.

Level 5 - Dog to recognise two scents (cloves and gun oil) and find 9 out of 12 possible scented articles in both interior and exterior searches.

Level 6 - Dog to recognise three scents (cloves, gun oil, truffle oil) and find 9 out of 12 possible scented articles in both interior and exterior searches.

Level 7 - Dog to recognise three scents (cloves, gun oil, truffle oil) and find 9 out of 12 possible scented articles in both interior and exterior searches. Each search area to include a decoy, unscented, article.

What level of scent-work training has your dog achieved? (equivalent to Mantrailing UK criteria Levels).

Mantrailing UK Level 1 - single blind trail, 200-400m trail with 1 change of direction, 30 mins - 1 hour aged trail with 1 target person.

Mantrailing UK Level 2 - double blind trail, 400-600m trail with 2 changes of direction, 1-2 hour aged trail with 1 target person.

Mantrailing UK Level 3 - double blind trail, 600-1000m trail with 2 changes of direction, 2-4 hour aged trail with 1 target person 1 decoy.

Does your dog have any formal background in agility training? *

Yes

No

What level of agility training has your dog received?

Beginner - Can sequence some obstacles, but not currently doing all obstacles at full height.

Pre-competition - Can complete all obstacles but has not competed in any events.

Competition - Has competed in independent shows with all obstacles at full height but has not participated in KC or UKA competition.

Competition - Competing at KC Grade 1-3 or UKA beginner.

Competition - Competing at KC Grade 4-5 or UKA novice.

Competition - Competing at KC Grade 6-7 or UKA senior.

Competition - Competing at KC championship or UKA champion.

Tables S1 to S5 include the data used for analysis.

Table S1: Background Data, training experience, eye contact duration and number of stress signals (over 60 seconds). Final comments indicates whether the dogs were included in the analysis of A not B and Detour data. Those with a ‘no’ indication did not pass familiarisation stages due to over-stimulation or distraction.

**I.D**	**Sex**	**Neuter status**	**Age**	**Breed**	**Breed type**	**# Disciplines**	**Combined training level**	**Primary discipline**	**Eye contact (sec)**	**# Stress signals**	A not B Analysis	Detour analysis
1	Male	Neutered	> 8 years	Springer Spaniel	Gundog	3	9	Obedience	No video	No video	yes	yes
2	Female	Entire	2 - 4 years	Labrador Retriever	Gundog	2	4	Scent	No video	No video	yes	yes
3	Female	Entire	6 - 8 years	Labrador Retriever	Gundog	2	8	Agility	No video	No video	yes	yes
4	Male	Neutered	6 - 8 years	Sheepdog	Pastoral	2	9	Agility	6.16	13	yes	yes
5	Female	Neutered	6 - 8 years	Chihuahua x Jack Russell	Other	3	6	Agility	0	5	yes	yes
6	Male	Entire	2 - 4 years	Cardigan corgi	Pastoral	3	4	Obedience	3.95	2	yes	yes
7	Female	Entire	< 12 months	Border collie	Pastoral	0	0	N/A	26.32	1	yes	yes
8	Male	Neutered	1 - 2 years	Welsh sheepdog	Pastoral	2	7	Agility	1.63	7	yes	yes
9	Female	Neutered	2 - 4 years	Lurcher	Other	2	7	Agility	0	9	yes	yes
10	Male	Entire	4 - 6 years	Boston Terrier	Other	2	5	Obedience	0	1	no	yes
11	Female	Entire	2 - 4 years	Rottweiler	Other	2	6	Obedience	15.56	7	yes	yes
12	Male	Neutered	> 8 years	Rottweiler	Other	2	7	Obedience	28.49	4	yes	yes
13	Male	Neutered	4 - 6 years	Labrador Retriever	Gundog	0	0	N/A	19.85	3	yes	yes
14	Female	Entire	1 - 2 years	Dobermann	Other	0	0	N/A	1.98	8	yes	yes
15	Female	Entire	2 - 4 years	Dobermann	Other	1	2	Scent	2.56	2	no	no
16	Female	Entire	< 12 months	Welsh sheepdog	Pastoral	1	1	Obedience	5.31	5	no	no
17	Male	Entire	< 12 months	Huntaway x border collie	Pastoral	1	1	Obedience	4.58	13	yes	yes
18	Male	Entire	2 - 4 years	Cardigan Welsh Corgi	Pastoral	1	1	Obedience	0	2	yes	yes
19	Female	Neutered	> 8 years	Cardigan Welsh Corgi	Pastoral	2	4	Agility	10.47	6	yes	yes
20	Female	Neutered	> 8 years	Mixed terrier	Other	1	1	Scent	6.58	2	yes	yes
21	Female	Neutered	> 8 years	Sheepdog	Pastoral	2	4	Obedience	22.41	2	yes	yes
22	Female	Neutered	> 8 years	Border Collie Cross (mixed)	Pastoral	0	0	N/A	0.91	6	yes	yes
23	Female	Neutered	1 - 2 years	Border Collie	Pastoral	2	2	Scent	3.72	9	yes	yes
24	Male	Entire	2 - 4 years	Springer spaniel	Gundog	1	3	Scent	15.19	0	yes	yes

25	Female	Entire	6 - 8 years	Labrador Retriever	Gundog	2	4	Scent	54.23	14	yes	yes
26	Female	Entire	2 - 4 years	Labrador Retriever	Gundog	2	5	Scent	33.27	3	yes	yes
27	Female	Neutered	2 - 4 years	Foxhound X Huntaway	Pastoral	2	7	Obedience	34.43	7	yes	yes
28	Male	Neutered	4 - 6 years	Labrador	Gundog	3	5	Agility	0	5	yes	yes
29	Female	Neutered	6 - 8 years	Springer spaniel x poodle	Gundog	3	10	Obedience	13.3	7	yes	yes
30	Male	Neutered	2 - 4 years	Border collie x poodle	Pastoral	3	7	Obedience	35.36	17	yes	yes
31	Female	Entire	1 - 2 years	Golden retriever X	Gundog	1	4	Obedience	11.61	12	yes	yes
32	Female	Neutered	2 - 4 years	Welsh sheep dog	Pastoral	2	5	Obedience	0	0	yes	yes
33	Female	Neutered	2 - 4 years	Terrier X	Other	2	4	Obedience	0.82	5	yes	yes
34	Male	Neutered	2 - 4 years	Border Collie X Huntaway	Pastoral	2	3	Obedience	27.5	12	yes	yes
35	Female	Entire	< 12 months	Husky x Belgian Malinois	Pastoral	1	1	Scent	3.54	3	yes	yes
36	Female	Neutered	2 - 4 years	Husky x border collie	Pastoral	1	2	Scent	0	2	yes	yes
37	Female	Entire	2 - 4 years	Border Collie	Pastoral	3	5	Agility	38.96	3	yes	yes
38	Male	Entire	2 - 4 years	Springer spaniel	Gundog	2	3	Scent	6.01	6	yes	yes
39	Female	Neutered	1 - 2 years	Labrador X Pointer	Gundog	2	3	Scent	1.39	0	yes	yes
40	Female	Entire	2 - 4 years	Border collie	Pastoral	1	4	Obedience	1.32	1	yes	yes

Table S2: A not B trial data indicating success rate at each stage for the A not B trials.

ID	Passed familiarisation	Passed 'A' training stage	Trials to pass training	AnotB TEST Success 8/10	Trial 1st correct choice TEST	AnotB Category
1	Yes	Yes	5	Yes	2	PASS_TRAIN_PASS_TEST
2	Yes	Yes	5	No	4	PASS_TRAIN_FAIL_TEST
3	Yes	Yes	5	Yes	1	PASS_TRAIN_PASS_TEST
4	Yes	Yes	5	No	3	PASS_TRAIN_FAIL_TEST
5	Yes	Yes	5	No	1	PASS_TRAIN_FAIL_TEST
6	Yes	Yes	11	No	2	PASS_TRAIN_FAIL_TEST
7	Yes	Yes	5	Yes	2	PASS_TRAIN_PASS_TEST
8	Yes	Yes	5	Yes	2	PASS_TRAIN_PASS_TEST
9	Yes	Yes	5	Yes	1	PASS_TRAIN_PASS_TEST
10	No	N/A	N/A	N/A	N/A	N/A
11	Yes	Yes	5	Yes	1	PASS_TRAIN_PASS_TEST
12	Yes	Yes	5	Yes	1	PASS_TRAIN_PASS_TEST
13	Yes	Yes	5	No	2	PASS_TRAIN_FAIL_TEST
14	Yes	No	Failed	N/A	N/A	FAIL_TRAIN
15	No	N/A	N/A	N/A	N/A	N/A
16	No	N/A	N/A	N/A	N/A	N/A
17	Yes	No	Failed	N/A	N/A	FAIL_TRAIN
18	Yes	No	Failed	N/A	N/A	FAIL_TRAIN
19	Yes	Yes	5	No	6	PASS_TRAIN_FAIL_TEST
20	Yes	Yes	5	Yes	1	PASS_TRAIN_PASS_TEST
21	Yes	Yes	5	No	1	PASS_TRAIN_FAIL_TEST
22	Yes	Yes	5	No	No correct choice during TEST	PASS_TRAIN_FAIL_TEST
23	Yes	Yes	5	No	N/A	PASS_TRAIN_FAIL_TEST
24	Yes	Yes	5	Yes	1	PASS_TRAIN_PASS_TEST
25	Yes	Yes	5	Yes	1	PASS_TRAIN_PASS_TEST
26	Yes	Yes	5	No	1	PASS_TRAIN_FAIL_TEST
27	Yes	Yes	5	Yes	2	PASS_TRAIN_PASS_TEST
28	Yes	Yes	5	No	1	PASS_TRAIN_FAIL_TEST
29	Yes	Yes	6	Yes	1	PASS_TRAIN_PASS_TEST
30	Yes	Yes	6	Yes	1	PASS_TRAIN_PASS_TEST
31	Yes	Yes	5	No	1	PASS_TRAIN_FAIL_TEST

32	Yes	Yes	5	Yes	1	PASS_TRAIN_PASS_TEST
33	Yes	Yes	5	Yes	1	PASS_TRAIN_PASS_TEST
34	Yes	Yes	5	No	No correct choice during TEST	PASS_TRAIN_FAIL_TEST
35	Yes	No	Failed	N/A	N/A	FAIL_TRAIN
36	Yes	Yes	5	No	2	PASS_TRAIN_FAIL_TEST
37	Yes	Yes	5	Yes	1	PASS_TRAIN_PASS_TEST
38	Yes	Yes	5	Yes	1	PASS_TRAIN_PASS_TEST
39	Yes	Yes	8	Yes	1	PASS_TRAIN_PASS_TEST
40	Yes	Yes	5	No	4	PASS_TRAIN_FAIL_TEST

Table S3: A not B trial data including number of correct choices, preservative error rate and speed to select a cup (sec) for dogs that completed all 10 TEST trials. Dogs are excluded if they did not pass the familiarisation or training phase, or if they exhibited stress during the test phase.

ID	# correct	Perservative error rate	Trial 1	Trial 2	Trial 3	Trial 4	Trial 5	Trial 6	Trial 7	Trial 8	Trial 9	Trial 10	Average Speed
1	9	1	2.05	1.58	1.82	1.72	1.81	1.68	1.61	1.44	1.86	1.46	1.703
2	5	5	3.58	3.27	3.44	3.39	3.19	3.27	3.32	3.12	2.36	2.29	3.123
3	9	1	2.29	3.71	2.25	2.61	2.13	2.75	2.06	2.17	2.37	1.91	2.425
4	3	7	2	2.38	2.72	2.28	2.26	2.24	2.44	2.77	3.03	2.96	2.508
5	5	5	3.15	2.87	3.18	3.98	4.53	3.9	4.78	5.68	3.67	3.29	3.903
6	5	5	5.33	4.84	4.46	6	5.7	8.82	5.87	5.8	4.16	4.5	5.548
7	9	1	1.91	2.15	1.71	1.68	2.13	1.57	1.65	1.52	1.74	1.69	1.775
8	9	1	1.76	2.76	2.44	2.33	2.04	2.12	2.28	2.11	2.33	2.31	2.248
9	10	0	1.68	2.41	2.05	2.16	1.99	1.95	1.93	2.1	2.05	1.89	2.021
11	10	0	1.81	1.98	2.38	1.69	1.78	1.85	1.72	1.71	1.95	1.82	1.869
12	10	0	2.25	2.32	2.36	2.84	2.33	2.35	2.26	2.28	2.12	2.69	2.38
13	6	4	2.18	5.89	2.16	1.79	1.68	1.52	1.85	1.92	1.85	1.68	2.252
19	2	8	1.89	2.33	2.39	2.77	2.18	5.88	4.32	2.9	5.41	4.61	3.468
20	10	0	2.04	1.89	1.81	1.95	2.06	2.05	2.13	1.92	1.66	1.61	1.912
21	6	4	4.06	9.13	3.59	3.84	3.77	4.24	4.16	4.07	3.15	3.51	4.352
23	0	10	2.06	2.05	2.51	2.76	2.33	2.25	2.51	2.89	2.63	8.61	3.06
24	10	0	1.36	1.4	1.34	1.49	1.46	1.48	1.31	1.29	1.2	1.26	1.359
25	10	0	1.22	1.4	1.42	1.29	1.31	1.26	1.29	1.21	1.24	1.27	1.291
26	7	4	2.01	1.74	1.73	1.8	1.84	1.47	1.64	1.74	1.9	1.54	1.741
27	9	1	1.41	1.38	1.36	1.35	1.52	1.35	1.46	1.35	1.42	1.36	1.396
28	2	8	1.57	1.59	1.68	1.47	1.81	1.59	1.65	1.84	1.8	1.78	1.678
29	10	0	1.33	1.44	1.28	1.27	1.36	1.42	1.39	1.36	1.47	1.38	1.37
30	9	1	4.4	4.2	4.86	4.29	4.71	3.83	2.34	2.68	3.14	5.75	4.02
31	7	4	2.03	1.64	2.41	1.65	2.43	2.91	4.93	2.07	1.99	4.9	2.696
32	10	0	2.7	1.7	1.67	1.48	1.61	1.72	1.83	1.62	1.85	1.84	1.802
33	10	0	1.43	1.32	1.37	1.33	1.22	1.16	1.2	1.28	1.18	1.14	1.263
36	6	4	1.87	2.09	1.56	1.47	1.22	1.72	2.08	1.61	1.57	1.48	1.667
37	10	0	1.49	1.45	1.42	1.39	1.27	1.48	1.32	1.36	1.38	1.48	1.404
38	9	1	1.1	1.21	1.15	1.19	1.11	1.25	1.13	1.09	1.19	1.27	1.169
39	10	0	1.43	1.52	1.75	1.64	1.56	1.62	1.58	1.73	1.67	1.75	1.625
40	4	6	4.83	11.92	2.22	3.11	9.78	7.61	7.4	1.45	4.08	3.71	5.611

Table S4: Detour task showing success rate, number of trials completed and first trial on which dogs successfully detoured without error.

I.D	DT success (8/10 direct)	Trials completed	First direct trial
1	Fail	10	1
2	Fail	10	3
3	Fail	10	2
4	Fail	10	2
5	Fail	8	1
6	Fail	10	1
7	Success	10	3
8	Success	10	3
9	Fail	10	5
10	Fail	10	4
11	Success	10	1
12	Fail	10	4
13	Fail	10	4
14	Fail	4	1
15	No attempt	N/A	N/A
16	No attempt	N/A	N/A
17	Fail	10	2
18	Fail	10	1
19	Fail	10	1
20	Fail	10	6
21	Fail	3	No direct trials
22	Fail	3	1
23	Fail	5	No direct trials
24	Fail	10	5
25	Success	10	1
26	Success	10	3
27	Fail	3	1
28	Fail	10	No direct trials
29	Success	10	1
30	Success	10	1
31	Success	10	1

32	Success	10	3
33	Success	10	1
34	Success	10	1
35	Fail	10	N/A
36	Success	10	1
37	Fail	10	6
38	Success	10	1
39	Success	10	1
40	Fail	10	6

Table S5: Detour task trial data only including dogs that completed all 10 detour task trials, including direct detour rate, error rate and detour time (sec).

I.D	# Direct trials	Contact errors	Path errors	Total errors	Trial 1	Trial 2	Trial 3	Trial 4	Trial 5	Trial 6	Trial 7	Trial 8	Trial 9	Trial 10	Average Speed
1	2	8	8	16	2.76	5.39	4.96	4.47	3.65	9.44	17.39	4.7	9.71	3.51	6.598
2	6	2	4	6	7.28	5.36	3.37	3.21	3.46	3.28	3.01	3.36	2.76	3.32	3.841
3	7	1	3	4	4.12	2.7	2.77	2.71	2.36	2.37	2.73	2.59	2.5	2.52	2.737
4	7	1	3	4	22.37	4.56	6.15	3.86	3.52	3.79	3.58	3.32	3.54	3.29	5.798
6	6	1	4	5	7.23	6.74	8	22.73	8.2	14.17	6.46	23.95	5.68	8.95	11.211
7	8	2	2	4	6.52	3.41	2.86	3.05	2.79	2.84	2.79	2.79	2.42	2.8	3.227
8	8	2	2	4	11.36	4.87	4.07	2.97	2.82	2.83	2.86	2.67	2.69	2.55	3.969
9	6	2	4	6	10.79	8.45	2.75	2.72	2.68	2.22	1.79	2.72	2.71	2.85	3.968
10	7	0	3	3	6.95	33.74	20.02	3.5	2.85	2.76	2.57	2.59	3.22	2.91	8.111
11	9	4	0	4	2.45	2.76	2.81	3.18	2.85	2.79	3.19	3.21	4.78	4.21	3.223
12	5	3	5	8	11.59	6.81	4.28	3.02	4.42	3.85	3.24	3.71	3.98	3.43	4.833
13	7	0	3	3	14.94	39.2	4.85	3.25	3.29	3.26	3.14	2.97	3.11	3.8	8.181
17	6	0	4	4	5.02	5.26	28.93	3.66	5.58	3.8	7.8	8	5.64	5.24	7.893
18	3	0	7	7	2.55	2.97	5.3	3.14	24.01	38.94	17.49	7.97	30.91	21.57	15.485
19	5	0	5	5	5.26	35.53	36.77	36.81	5.83	16.12	34.76	4.03	3.46	3.33	18.19
20	5	0	5	5	7.02	10.65	3.71	3.03	3.79	3.91	3.28	3.39	4.06	3.69	4.653
24	4	1	6	7	4.54	3.03	3.16	7.4	3.04	4.53	7.19	2.47	2.77	2.89	4.102
25	8	0	2	2	3.04	3.22	3.3	4.94	3.31	3.26	3.32	3.6	3.79	3.6	3.538
26	8	0	2	2	5.54	9.04	3.79	3.88	3.44	3.3	3.43	3.92	3.56	3.79	4.369
28	0	0	10	10	26.39	5.89	2.93	2.86	2.58	2.85	2.74	2.96	3.11	3.72	5.603
29	10	0	0	0	3.03	2.37	2.52	2.73	2.53	2.76	2.64	2.82	2.71	2.91	2.702
30	10	0	0	0	2.43	2.94	2.61	2.62	2.37	2.6	2.66	2.82	2.95	2.68	2.668
31	9	0	1	1	3.04	2.47	2.61	2.49	2.69	2.41	4.87	2.29	2.35	2.61	2.783
32	8	0	2	2	5.87	5.02	2.97	2.91	3.03	2.9	2.94	3.06	3.08	3.02	3.48
33	10	0	0	0	2.42	2.53	2.4	2.52	2.53	2.46	2.47	2.43	2.35	2.51	2.462
34	10	1	0	1	2.46	2.56	2.81	2.78	2.31	2.49	3.11	2.87	2.44	2.8	2.663
35	0	0	10	10	7.01	5.79	4.68	22.15	9.03	4.7	5.35	10.3	5.33	6.67	8.101
36	9	0	1	1	2.6	2.38	2.75	2.71	2.44	3.06	2.96	2.77	4.84	4.41	3.092
37	5	0	5	5	18.59	23.97	7.38	3.25	7.98	2.55	2.57	2.67	2.32	2.91	7.419
38	10	0	0	0	3.81	2.97	2.91	2.61	2.51	2.52	2.49	3.05	2.92	2.51	2.83
39	8	0	2	2	3.17	3.53	2.97	2.82	3.18	2.9	2.38	3.04	2.99	3.01	2.999
40	2	4	8	12	12.87	6.22	7.03	8.61	12.1	3.5	6.76	6.84	3.5	4.95	7.238
